# High-resolution quantitative mapping of extracellular pH by ratiometric MRI with iron chelates in a tumor mouse model

**DOI:** 10.1007/s11547-025-02020-z

**Published:** 2025-05-20

**Authors:** Honglan Mi, Philipp Boehm-Sturm, Akvile Haeckel, Ying Li, Susanne Mueller, Fei Ni, Harald Kratz, Marco Foddis, Jing Xie, Eyk Schellenberger

**Affiliations:** 1 Department of Radiology, Charité - Universitätsmedizin Berlin, corporate member of Freie Universität Berlin and Humboldt-Universität zu Berlin, Charitéplatz 1, 10117 Berlin, Germany; 2 Charité 3R – Replace | Reduce | Refine, Experimental Imaging at the Charité for 3R (EPIC3R), Charité - Universitätsmedizin Berlin, Charitéplatz 1, 10117 Berlin, Germany; 3 Department of Experimental Neurology and Center for Stroke Research Berlin, Charité - Universitätsmedizin Berlin, Charitéplatz 1, 10117 Berlin, Germany; 4 Charité Core Facility Experimental MRIs, Charité - Universitätsmedizin Berlin, Charitéplatz 1, 10117 Berlin, Germany

**Keywords:** pH mapping, Low molecular weight iron-based contrast agents (IBCA), Breast tumor, Molecular imaging of pH, RpH-MRI

## Abstract

**Purpose:**

The aim of this study was to generate quantitative extracellular pH maps of tumors using a combination of a pH-sensitive iron chelate-based contrast agent (IBCA) and a pH-insensitive IBCA for concentration measurement, which we termed ratiometric pH magnetic resonance imaging (RpH-MRI).

**Methods:**

The pH-sensitive IBCA of ethylenediamine-trans-cyclohexane diamine tetraacetic acid (Fe-en-tCDTA) was synthesized, along with the pH-insensitive IBCAs of trans-cyclohexane diamine tetraacetic acid (Fe-tCDTA) and diethylenetriamine-N,N,N′,N″,N″-pentaacetic acid (Fe-DTPA). The pH-dependent T1 contrast effects of these chelates were compared in water and serum phantoms at 0.94 T, 3 T and 7 T. For in vivo pH mapping of tumors at 7 T, 4T1 breast cancer cells were inoculated subcutaneously into the flanks of the BALB/c mice. RpH-MRI was performed with two sequential intravenous applications: first a pH-insensitive IBCA, followed by the pH-sensitive IBCA at the same dose (0.25 or 0.5 mmol/kg) with an interval of either 30 or 60 min. Quantitative pH maps were generated by calculating T1, S_0_, and relative maximum enhancement maps of the two injections, together with pH-dependent T1-relaxivity parameters derived from in vitro measurements of the pH-sensitive IBCA and pH-insensitive control IBCA.

**Results:**

The T1 relaxivity (r1) of Fe-en-tCDTA was highly pH dependent, being approximately 2.7 times higher at pH 5.5 than at neutral pH, whereas Fe-DTPA and Fe-tCDTA showed stable r1 values between pH 5.5–7.4. In vivo, the time to maximum signal intensity (TMI) of the tumors of Fe-DTPA as control was comparable to that of Fe-en-tCDTA (2.57 ± 1.34 min vs. 2.683 ± 0.89 min, *p* = 0.7596, paired t test, 4 mice, 7 tumors) as well as for Fe-tCDTA as control versus Fe-en-tCDTA (3.30 ± 1.17 min vs. 3.627 ± 1.12 min, *p* = 0.2101, paired t test, 7 mice, 13 tumors), suggesting similar pharmacokinetics. The concentration distribution at TMI of the control chelates was assumed to be the same as that of the second injected Fe-en-tCDTA. The dynamic contrast enhanced MRI curve of the first injection of Fe-DTPA returned to baseline after 20–30 min, whereas Fe-tCDTA took 30–60 min to reach baseline. Calculated core and rim pH values were 6.512 ± 0.182 and 6.742 ± 0.121, respectively (*p* < 0.0001, paired t test, 11 mice, 20 tumors) with core areas showing lower chelate concentrations but higher T1 relaxivity; the mean tumor-wide pH value was 6.632 ± 0.140.

**Conclusion:**

Our results demonstrate the potential of high-resolution RpH-MRI based on pH-sensitive and pH-insensitive IBCAs for mapping tumor extracellular pH and concentration distribution.

**Supplementary Information:**

The online version contains supplementary material available at 10.1007/s11547-025-02020-z.

## Introduction

Decreased extracellular pH is associated with ischemic diseases, such as stroke, ischemic heart disease, renal disease, and especially cancer [1–4]. Tumors exhibit increased metabolic activity, leading to the generation of excess acid, which is rapidly released into the extracellular space, making it a promising target for monitoring [5].

Despite extensive efforts to develop pH-responsive agents for MRI, there are relatively few successful reports of tumor extracellular pH imaging using MRI in the literature. Examples include gadolinium complexes [6–8] and gadolinium-containing polymeric nanoparticles [9] with pH-sensitive switchable structures as well as manganese-based nanostructures that release paramagnetic Mn^2+^ ions in the acidic tumor microenvironment [10]. However, the practical utility of many nanoparticle-based agents is limited by their high uptake by the reticuloendothelial system organs, such as the liver and spleen, leading to slow and inefficient elimination [11]. The pH-sensitive gadolinium probe GdDOTA-4AmP, based on the relatively stable FDA-approved Gd-DOTA (Dotarem^®^, Guerbet) contrast agents, has been used for pH mapping and is, in part, the basis for our approach but has a relatively modest pH dependence of r1 and a relatively slow tissue clearance [7, 12].

While clinically approved gadolinium-based contrast agents (GBCAs) are eliminated relatively rapidly by renal clearance, the leakage of toxic Gd^3+^ ions from traces of the remaining complexes poses risks such as nephrogenic systemic fibrosis in patients with impaired renal function [13] and Gd^3+^ is retained in organs such as the brain, skin, and bone, e.g. 10 weeks after a single clinical dose in sheep [14–16]. A condition known as Gadolinium deposition disease (GDD) has been described in patients who present with a variety of symptoms [17]. In contrast, iron, an endogenous metal essential for multiple biological processes in every cell of the body, represents a promising alternative contrast agent owing to its biocompatibility and natural metabolism.

We have recently shown that low-molecular-weight IBCAs, in particular, iron(III)-trans-1,2-diaminocyclohexane-N,N,N′,N′-tetraacetic acid (Fe-tCDTA) and its derivatives, as T1 contrast agents, are comparable to GBCAs at only twice the dose in typical MRI applications such as dynamic contrast-enhanced MRI (DCE-MRI) and MR angiography [18, 19]. Based on Fe-tCDTA, we developed several new derivatives with improved properties, for example, T1 relaxivities of up to 6.8 mM^−1^ s^−1^ [18, 20]. One of the new iron complexes, iron(III)-ethylenediamine-trans-CDTA (Fe-en-tCDTA), exhibited pronounced pH dependence with a substantial increase in T1 relaxivity under acidic conditions, thus providing a strong T1 contrast effect in the slightly acidic pH range prevalent in many tumors.

**Fig. 1:**
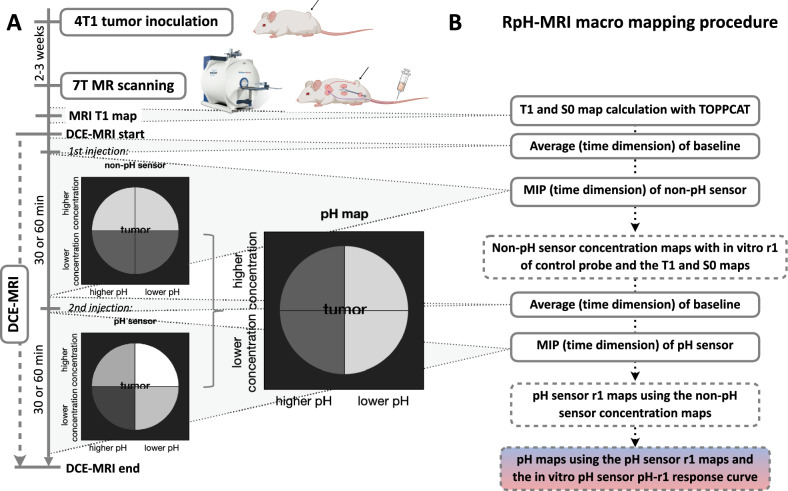
Study procedure and in vivo pH map generation by RpH-MRI. **A** Schematic illustration of tumor model setting and 7T MR scanning procedure, as well as the hypothesis of discriminating between concentration effects and pH effects by the RpH-MRI procedure using non-pH sensor and pH sensor iron complexes for in vivo tumor pH mapping. **B** Illustration of the macro computational procedure used to generate the pH maps. MIP—Maximum intensity projection

Our hypothesis of using Fe-en-tCDTA for pH mapping is that, given that the T1 contrast signal of the compound is influenced by its pH-dependent r1 and its concentration, it is possible to distinguish the pH and concentration components by combining the time-delayed application of the iron-based pH probe and a non-pH-sensitive iron complex with similar properties. This approach should allow for the calculation of approximate pH tissue maps (Fig. 1). This ratiometric pH MRI (RpH-MRI) should therefore provide new imaging biomarkers of tumor biology that could ultimately improve diagnostics and therapy monitoring.

## Materials and methods

### Synthesis of iron compounds

Fe-tCDTA, Fe-en-tCDTA, and iron(III)-diethylenetriamine-N,N,N′,N″,N″-pentaacetic acid (Fe-DTPA) were synthesized and characterized according to the literature references [18, 19]. These compounds were purified, sterile-filtered, and autoclaved. Detailed information is provided in the supplementary file.

### T1 relaxivity testing of phantoms

The T1 contrast effects of Fe-DTPA, Fe-tCDTA, and Fe-en-tCDTA were compared in MR imaging phantom experiments using water and fetal bovine serum (FBS; Gibco, ThermoFisher Scientific, USA, A5256701) as a blood-like environment. Additionally, Fe-en-tCDTA was tested in saline and saline with albumin (albumin concentration: 2.12 g/100 mL, matching that in FBS). All phantoms were loaded into glass NMR tubes (5 mm outside diameter; Wilmad-Lab Glass Company) and were maintained at 37 °C ± 1 °C by recirculating warm water in a specific holder. The Fe^3+^ concentrations used were 0, 0.5, 2.5, and 5 mmol/L. Measurements at 0.94 T NMR relaxometer (Bruker Minispec mq 40, Karlsruhe, Germany) were done according to the manufacturer’s instructions. Measurements at a 3 T MRI device MAGNETOM Lumina (Siemens Healthineers, Forchheim, Germany) used standard 2D spin echo sequences with different repetition times (TRs) of 100, 150, 300, 600, and 1000 ms to measure T1 times and calculate T1 relaxivities. The echo time (TE) was 13 ms, with a matrix of 256 × 256, field of view of 75 × 75 mm^2^ and slice thickness of 5 mm. Imaging parameters at the 7 T small-animal MR imaging unit (BioSpec 70/20 USR, Bruker, Ettlingen, Germany), detailed in Table 1, were consistent with those used in subsequent in vivo MRI studies.

**Table 1:** MRI acquisition parameters at 7 T

Parameters	T2WI	VFA T1 Map	DCE
Pulse sequence	RARE 2D	FLASH 3D	FLASH 3D
Tx/Rx radiofrequency coil setup	Volume	Volume	Volume
Field of view (mm)	36 × 48	36 × 48 × 24	36 × 48 × 24
Image matrix	180 × 240	90 × 120 × 60	90 × 120 × 60
No. of sections	20	60	60
Section thickness (mm)	1	24	24
Repetition time (msec)	1600	13	13
Echo time (msec)	16	2.4	2.4
Flip angle (degrees)	90	2, 5, 15, 30, 50, 70	15
Averages	4	1	1
No. of repetitions	1	1	314
Acquisition time	2 min 20 s	2 min 20 s	1 or 2 h and 2 min 27 s

2D, two-dimensional; 3D, three-dimensional; RARE, rapid acquisition with relaxation enhancement; FLASH, fast low-angle shot; VFA, variable flip angle; DCE, dynamic contrast enhancement

### Cell culture and cell viability assay

All cell culture media and solution were from Gibco, ThermoFisher Scientific, USA. The 4T1 breast tumor cell line (ATCC, USA, RRID: CVCL_0125) was cultured in RPMI-1640 medium supplemented with 10% heat-inactivated FBS and 1% penicillin/streptomycin (Gibco, ThermoFisher Scientific, USA). The BRL-3A hepatocytes (ATCC, USA, RRID: CVCL_0606) were cultured in DMEM medium supplemented with 10% heat-inactivated FBS and 1% penicillin/streptomycin. Both cell lines were maintained at 37℃ in a humidified atmosphere containing 5% CO_2_.

To evaluate the cytotoxicity of Fe-en-tCDTA, BRL-3A and 4T1 cells were incubated with various concentrations (0, 5, 10, 20, and 40 mM) of Fe-en-tCDTA separately in 96-well flat-bottom plates for 24 h. The cells were seeded at a density of 10^4^ cells/well. Cell viability was assessed using the MTT Assay kit (Abcam, Cambridge, UK) according to the manufacturer's instructions. Cell survival was calculated as a percentage of the blank control. Each experiment was performed in triplicate and was repeated three times. The percentage cytotoxicity was calculated using the following equation, based on the corrected absorbance: cell viability [%] = sample/control × 100.

### Tumor inoculation

All animal procedures were performed in accordance with the guidelines of the Institutional Animal Care and Use Committee of Berlin (Landesamt für Gesundheit und Soziales (LAGeSo) Berlin, approval number G0148/21). During the exponential phase of growth, 4T1 cells were harvested and cryopreserved in FBS containing 10% DMSO at a density of 10^6^ cells/vial. Each vial of thawed cells was washed with growth medium and resuspended in Dulbecco's balanced salt solution (Gibco, ThermoFisher Scientific, USA) to achieve a concentration of 10^6^ cells/ml. Subsequently, 100 µL of the cell suspension was inoculated subcutaneously into the bilateral flanks of the 8- to 12-week-old female BALB/c mice (Janvier Labs, France. RRID: MGI: 2683685) with weight 19 to 28 g. Tumor growth was monitored for approximately 2–3 weeks until the tumor diameter reached at least 5 mm. The tumor mice were randomly assigned to receive the two different control substances for in vivo MRI experiments.

### In vivo MRI experiments and study design

The experimental procedure is illustrated in Fig. 1. Experiments were conducted using a 7 T device (BioSpec 70/20 USR, Bruker, Germany). A 35 mm diameter volume coil (RAPID Biomedical GmbH, Germany) was used for signal transmission and reception. Animals were anesthetized with 1–2% (v/v) isoflurane in a mixture of 30% O_2_ and 70% N_2_O and then placed on a water bath heated warming mat. Throughout the experiment, the body temperature and respiration rate were continuously monitored with a rectal probe and pressure-sensitive pad underneath the thorax, respectively, using an MR-compatible monitoring system (SA Instruments, Stony Brook, NY, USA). The temperature was maintained at 37–37.5 °C using recirculating warm water.

Following a localizer scan and T2-weighted (T2w) anatomical imaging, T1-weighted spoiled gradient echo (SPGR) imaging using multiple flip angles (2°, 5°, 15°, 30°, 50°, and 70°) was performed to generate T1 and S_0_ maps [21] (see Macro script 1 provided in the Supplementary material). The signal $S\left(\alpha \right)$ was fitted as follows:

1 $$S\left(\alpha \right)={S}_{0}\left(1-{e}^{-TR/T1}\right)\text{sin}\left(\alpha \right)/(1-\text{cos}(\alpha ){e}^{-TR/T1})$$

where $TR$ is the repetition time, $\alpha$ is the flip angle, and ${S}_{0}$ is the longitudinal equilibrium magnetization.

Subsequently, pH-MRI was performed. The pH-insensitive (either Fe-DTPA or Fe-tCDTA) and pH-sensitive Fe-en-tCDTA were sequentially injected via the tail vein at intervals of 30 min or 1 h. The IBCA injections were administered at a dose of 0.5 mmol/kg or 0.25 mmol/kg at an infusion rate of 3–5 µl/s over a total injection time of 20 s. Imaging parameters are provided in Table 1. The DCE curves and time to maximal intensity (TMI) were measured to assess the pharmacokinetics of these IBCAs. All values were measured in the most stable and maximal sections of the aorta and tumor.

### Tumor pH mapping procedure

The relaxation rate ${R}_{1}\left(t\right)$ maps for each time point $t$ of the dynamic images were calculated using the standard DCE-MRI procedure [22] as follows:

First, the equilibrium magnetization M_0_ map was calculated via

2 $${M}_{0}={S}_{0}\frac{1-\text{cos}\left(\theta \right){E}_{10}}{\text{sin}\left(\theta \right)\left(1-{E}_{10}\right)}, {E}_{10}={e}^{\left(-{T}_{R}/{T}_{10}\right)}$$

where ${S}_{0}$ is the baseline steady-state DCE signal derived by averaging pre-contrast DCE images, $\theta$ is the flip angle of the DCE scan, and ${T}_{10}$ is the pre-contrast longitudinal relaxation time.

Second, the relaxation rate ${R}_{1}\left(t\right)$ was calculated via

3 $${R}_{1}\left(t\right)=-\frac{1}{{T}_{R}}\text{ln}\left(\frac{1-\left(A\left(t\right)+B\right)}{1-\text{cos}\left(\theta \right)\left(A\left(t\right)+B\right)}\right), A\left(t\right)=\frac{S\left(t\right)-{S}_{0}}{{M}_{0}\text{sin}\left(\theta \right)}, B=\frac{1-{E}_{10}}{1-\text{cos}\left(\theta \right){E}_{10}}$$

where $S\left(t\right)$ is the DCE signal at time $t$.

Finally, the extracellular pH for each pixel was calculated according to a previously described method [7].

4 $$pHe=logIC50+\text{log}\left(\frac{Top-r1 map}{r1 map-Bottom}\right)/HillSlope$$

where $r1 map$ values are derived from the r1 map of the Fe-en-tCDTA (pH sensor) in vivo, and other parameters are from the pH sensor in vitro curve ($Top$ and $Bottom$ are plateaus in the units of the Y axis). $IC50$ is the concentration of agonist that gives a response halfway between the Bottom and Top. $HillSlope$ describes the steepness of the family of curves.).

After the first injection of the control probe (Fe-tCDTA or Fe-DTPA), a maximum intensity projection (MIP) image was obtained. Together with the r1 of the control probe from in vitro testing, its corresponding concentration map was calculated (see Macro script 2 provided in the Supplementary material). These concentration maps were considered and set equal to the concentration map of the pH sensor (second injection); with the combination of the concentration map and the MIP image of the pH sensor, the T1 relaxivity map of the pH sensor was generated, and the pH map was obtained using Eq. 4.

### Statistical analysis

The MRI File Manager (Bruker) (version 1.2) was used to import the data to the FIJI software (RRID:SCR_002285. https://imagej.net/ij/plugins/mri-file-manager/index.html). For each animal, the T1, S_0_, concentration, r1, and pH maps were calculated using the FIJI plugin TOPPCAT (https://sites.duke.edu/dblab/TOPPCAT/) and FIJI macro scripts, which are provided as supplementary files. The selection of regions of interest (ROI) for statistical analysis was performed using FIJI as follows: In the MRI signal slices with the largest tumor area, the whole tumor ROI was selected. From this, the core region ROIs were generated by downscaling the whole tumor ROI using the command “edit > scale > selection > scale: 0.7”. The rim ROI was created by applying the “XOR” command to the entire tumor ROI and core ROI. These ROIs were applied to the pH maps and statistically analyzed.

Comparisons of in vivo DCE curves, TMI, in vitro phantom T1 relaxivities, and MTT assay results were performed using GraphPad Prism 10 software (Boston, USA. RRID:SCR_002798). To assess the statistical significance of the difference between the first and second injections of agents, a two-tailed t-test for dependent samples was employed. The hypothesis was examined using an independent two-tailed t-test (unpaired t-test to assess the difference between Fe-DTPA group and Fe-tCDTA group). Additionally, the Pearson Correlation analysis was conducted to evaluate the correlation between the pH and tumor size. Statistical values are presented as mean ± SD. Statistical significance was established at *p* < 0.05. As this was a pilot study, a formal power calculation was not conducted.

MarvinSketch (Verion 24, Chemaxon, https://www.chemaxon.com) were utilized for drawing, displaying, and characterizing chemical structures, substructures, and reactions.

## Results

### Properties and T1 relaxivities of Fe-en-tCDTA and the control probes

**Fig. 2:**
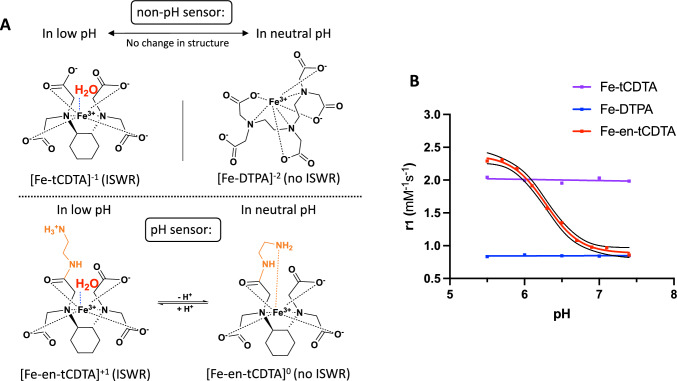
Chemical structures and T1 relaxivity comparison of iron-based contrast agents in vitro at 7 T. **A** Illustrations show molecular structures when pH changes. ISWR: inner-sphere water relaxation. **B** All phantoms of iron compounds were prepared with fetal bovine serum as a blood-like environment and imaged at 7 T for T1 mapping at 37 °C. The concentrations of Fe^3+^ were 0, 0.5, 2.5, 5 mmol/L. The T1 relaxivity (r1) of Fe-en-tCDTA was about 2.7 times higher at pH 5.5 than at neutral pH. The dotted lines present 95% CI values. Fe-DTPA and Fe-tCDTA exhibited stable r1s from pH 5.5–7.4

The molecular structures and T1 relaxivity values of Fe-DTPA, Fe-tCDTA, and Fe-en-tCDTA are shown in Fig. 2A and summarized in Table 2 and Fig. S1. All investigated IBCAs have a linear structure and similar molar masses, ranging from 398 to 446 g/mol. Fe-en-tCDTA is neutral, whereas Fe-tCDTA carries a single negative charge, and Fe-DTPA possesses two negative charges. The iron chelates remained stable without precipitation, such as iron hydroxide formation, for a period of at least one and a half years at room temperature, at concentrations of approximately 500 mM, and neutral pH. The r1 values of all investigated iron chelates were strongest at 3 T, and then 7 T, followed by 0.94 T (Fig. S1). All relationships between the relaxation rate 1/T1 and concentration were linear (Fig. S2).

**Table 2:** Comparison of low molecular iron compounds in vitro at pH 7.4

Compounds	Molar mass (g/mol)	r1 (L mmol^−1^ sec^−1^) at pH 7.4 (37 °C)					
		0.94 T		3 T		7 T	
		In water	In serum	In water	In serum	In water	In serum
Fe-en-tCDTA	440.42	0.76	0.84	0.96	1.06	0.86	0.86
Fe-tCDTA	398.35	1.40	1.58	2.03	2.26	1.62	1.98
Fe-DTPA	445.35	0.71	0.80	0.92	1.00	0.81	0.85

In the case of Fe-tCDTA, one coordination side is available for the exchangeable coordination of water molecules (Fig. 2A), allowing efficient inner sphere relaxation, irrespective of pH, contributing to its relatively high r1 (Figs. 2B, S1). In contrast, Fe-DTPA lacks a water coordination site, which accounts for its relatively low relaxivity at all pH levels. However, Fe-en-tCDTA also lacks a water coordination site at neutral pH. It is most likely that as the pH decreases, the protonation of the terminal amino group prevents its coordination to iron, thereby releasing the position for water coordination; this results in a significant pH dependence, being approximately 2.7 times higher at pH 5.5, compared to neutral pH. At neutral pH, the r1 of Fe-DTPA was comparable to that of Fe-en-tCDTA (0.8494 vs. 0.8583, mM^−1^S^−1^), whereas the r1 of Fe-tCDTA was more than twice that of Fe-en-tCDTA (1.9834 vs. 0.8583, mM^−1^S^−1^) in serum at 7 T. T1-weighted SPGR images with different flip angles and T1 maps are shown in Figure S3. T1 relaxivity measurements are presented in Table 2 and Fig. S1. To control for potential variance between T1 mapping sequences, we compared the variable flip angle (VFA) with the variable repetition time (VTR) mapping. The calculated r1 values of Fe-en-tCDTA at low pH based on VFA were slightly higher compared to the VTR measurements (Fig. S4) and were used as a pH-reference curve, and thus in vivo T1 mapping was also performed with VFA. Furthermore, no significant differences in r1s were observed for Fe-en-tCDTA in saline, saline with albumin, and FBS at pH 5.5, 6.5, and 7.4, indicating that Fe-en-tCDTA does not bind to albumin in the blood (Fig. S5).

### Toxicity analysis

The biocompatibility of Fe-en-tCDTA was initially evaluated by exposing BRL-3A and 4T1 cells to the complex at various Fe^3+^ concentrations for 24 h, followed by the analysis of cell viability using the MTT assay. As depicted in Figure S6, Fe-en-tCDTA at concentrations of up to 20 mM had an acceptable effect on cell viability, maintaining viability above 80% in both cell lines. These results indicated that Fe-en-tCDTA exhibited low cytotoxicity.

### In vivo pH-mapping by RpH-MRI in a 4T1 breast cancer model

Figure S7 shows the preparatory experiments for the selection of suitable intervals for RpH-MRI. Figure S7 A presents the signal intensity changes over time in the aortas of 4 mice, each administered different combinations of Fe-DTPA, Fe-tCDTA, and Fe-en-tCDTA. The injections were performed at intervals of 30 or 60 min. Among these, Fe-DTPA returned to baseline within 30 min, whereas Fe-en-tCDTA and Fe-tCDTA required more than 30 min but less than 1 h to reach the baseline levels. The signal peak and shape of the first injection of Fe-DTPA were similar to those of the second injection of Fe-DTPA, indicating good accuracy of the sequential injection method. The signal peaks were comparable between Fe-DTPA and Fe-en-tCDTA, while the signal peak of Fe-tCDTA was approximately twice that of Fe-en-tCDTA when using the same concentrations, consistent with the in vitro relaxivities in serum and water. Figure S7B–D present the signal intensity changes over time in the tumors of 3 mice (2 tumors each). The signal height and shape between the sequential injections of Fe-DTPAs were similar, corresponding to their behavior in the aorta. However, the signal peak ratio for the second injection of Fe-en-tCDTA to the first injection of the control probe (either Fe-DTPA or Fe-tCDTA) was higher than that in the aortas. Despite limited statistical data in the preparatory experiments, this difference is likely due to the acidic tumor microenvironment, which should increase the T1 relaxivity of the pH sensor (Fe-en-tCDTA), resulting in an increased signal peak.

**Fig. 3:**
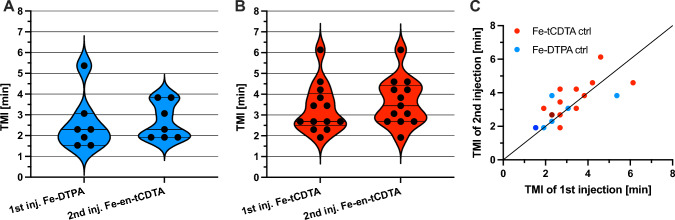
Comparison of time to maximum intensity (TMI) values. **A** No significant difference was observed between the TMI values of Fe-DTPA as first and Fe-en-tCDTA as second injections (2.57 ± 1.34 min vs. 2.683 ± 0.89 min, *p* = 0.7596, 4 mice, 7 tumors, mean ± SD paired t-test). Violin plots with median and quartiles. **B** No significant difference was observed between the TMI values of Fe-tCDTA as first and Fe-en-tCDTA as second injections (3.30 ± 1.17 min vs. 3.627 ± 1.12 min, *p* = 0.2101, 7 mice, 13 tumors, mean ± SD, paired t-test). **C** In addition, TMI values of double injections from each tumor are shown. The black line indicates equal TMI. Dark dots are two dots at the same times

For the pH mapping studies, 4 mice (7 tumors) received a sequential injection of 1st Fe-DTPA and 2nd Fe-en-tCDTA, and 7 mice (13 tumors) received 1st Fe-tCDTA and 2nd Fe-en-tCDTA. To assess the pharmacokinetics of the first injection of the control probes and the second injection of the pH sensor, their TMIs were calculated and compared (Fig. 3). As a result, the pharmacokinetics of the first and second injections could be assumed to be similar, particularly regarding their wash-in kinetics in the tumor area and the concentration distribution of compounds at the signal peak.

**Fig. 4:**
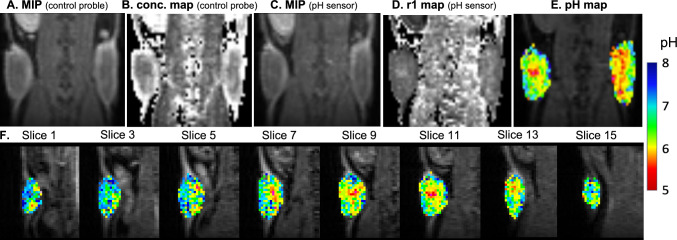
An example of RpH-MRI of Fe-tCDTA (1st injection as control) and pH-sensor (2nd injection) mapping the tumor pHs. **A** T1-weighted image with maximum intensity projection (MIP) of the first injection (control probe); **B** concentration (conc.) map of the first injection (control probe); **C** T1-weighted image with MIP of the second injection (pH sensor); **D** T1 relaxivity (r1) map of the second injection (pH sensor); **E** pH map; **F** demonstrates pH maps of various slices of the left tumor. All shown images are in coronal orientation and dose is 0.25 mmol/kg

**Fig. 5:**
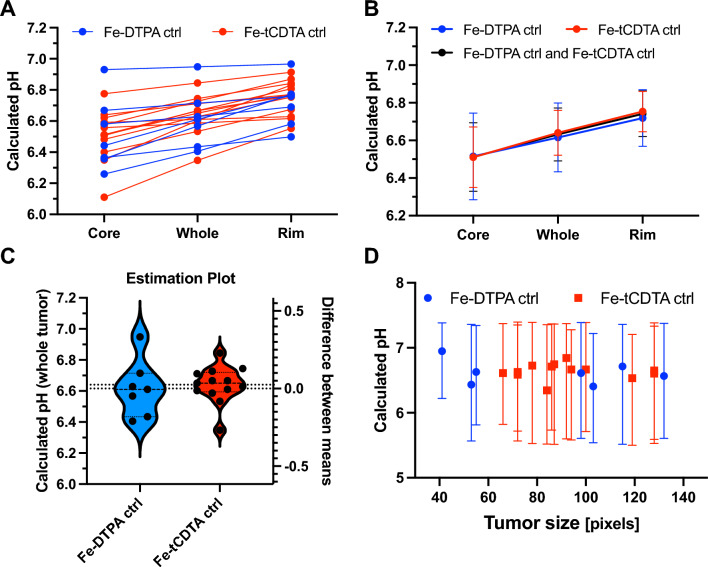
Comparison of pH distribution. **A** Individual mean pH values of the tumor core, rim, and whole tumor areas. **B** The average of the mean pH values of the core is significantly lower than the average of the rim (6.512 ± 0.182 vs. 6.742 ± 0.121, 11 mice, 20 tumors, *p* < 0.0001, paired t test); the average pH of the whole tumor was 6.632 ± 0.140. **C** There is no significant difference of the pH values when using Fe-DTPA vs. Fe-tCDTA as control agent (*p* = 0.7188, unpaired t test); **D** No correlation was found between the tumor size (of the slices with maximal area in pixels for each tumor) and the pH value (11 mice, 20 tumors, *p* = 0.4333, Pearson correlation)

Figure 4 shows an example of the tumor pH mapping procedure using RpH-MRI. The pH ranges of the left and right tumors were 5.608–7.372 and 5.504–7.310, respectively. The pH of the core area was lower than that of the rim area in the pH maps, as corroborated by the data from 11 mice in this study (Figs. 5, S8). The mean pH values of the core and rim of the axial panes (Figure S8) were 6.512 ± 0.182 and 6.742 ± 0.121, respectively (*p* < 0.0001, paired t-test). The mean pH value of the entire tumor was 6.632 ± 0.140. The core regions exhibited lower concentrations of chelates but higher r1 values, which is most likely attributable to poor perfusion. No correlation was found between tumor size and pH (*p* = 0.4333; Pearson correlation).

**Fig. 6:**
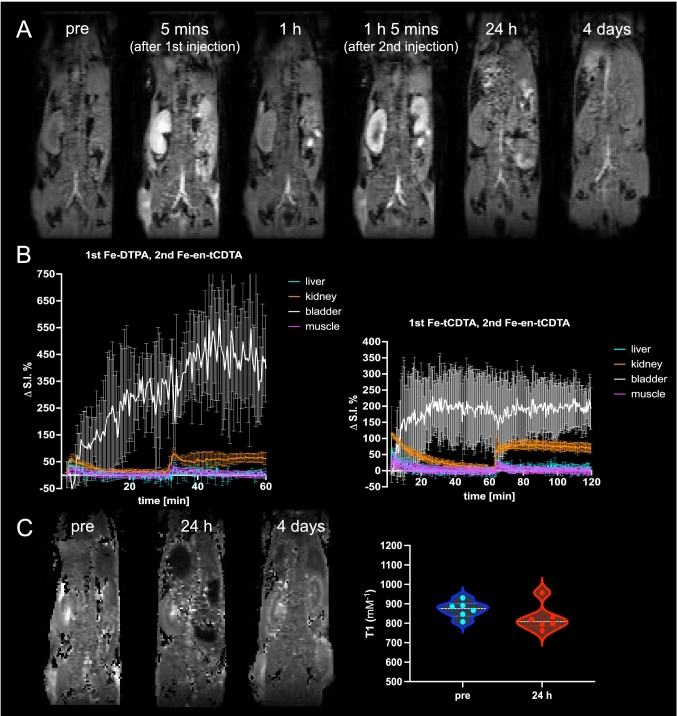
The clearance of IBCAs in vivo. **A** Representative DCE T1-weighted images at different time points after sequential injection of 1st Fe-tCDTA and 2nd Fe-en-tCDTA at dose 0.25 mmol/kg. The contrast agents are quickly excreted though the kidneys and after 24 h completely cleared from the kidney. **B** DCE curves in selected organs showing clearance from peripheral organs and accumulation of the agent in the bladder (n = 3 mice for group 1st Fe-DTPA and 2nd Fe-en-tCDTA at dose 0.5 mmol/kg; n = 4 mice for group 1st Fe-tCDTA and 2nd Fe-en-tCDTA at dose 0.25 mmol/kg). **C** Representative T1 maps before, 24 h after, and 4 days after sequential injection of 1st Fe-tCDTA and 2nd Fe-en-tCDTA at dose 0.25 mmol/kg. There was no significant difference among T1 values on the areas of both sides of renal parenchyma between before and 24 h after injections (n = 3 mice). RpH-MRI: ratiometric dynamic contrast-enhanced magnetic resonance imaging

Figure 6A illustrates the temporal biodistribution and clearance of iron compounds in vivo, specifically 1st Fe-tCDTA and 2nd Fe-en-tCDTA, administered at a dose of 0.25 mmol/kg. Injection of the agents into mice resulted in significant image enhancement of the kidneys. At 24 h post-injection, the signal intensity in the kidneys returned to baseline levels as pre-injection. Moreover, Fig. 6B shows excretion of the IBCAs predominantly through the kidneys and bladder, indicating rapid renal clearance (1st Fe-DTPA and 2nd Fe-en-tCDTA at a dose of 0.5 mmol/kg (n = 3); 1st Fe-tCDTA and 2nd Fe-en-tCDTA at a dose of 0.25 mmol/kg (n = 4)). Minimal amounts of the agents were processed by the liver, with even less detected in the muscle area, which was almost entirely cleared after 2 h. We did not observe any indication of excretion in the gall bladder or intestine. Additionally, Fig. 6C shows T1 maps before, 24 h after, and 4 days after the sequential injection of 1st Fe-tCDTA and 2nd Fe-en-tCDTA at a dose of 0.25 mmol/kg. There was no significant difference among the T1 values of both sides of the renal parenchyma before and 24 h after injections (n = 3).

## Discussion

Based on recently developed IBCA derivatives [18], the objective of this study was to exploit the pH-dependent T1 relaxivity of the Fe-en-tCDTA complex for pH mapping in tumors in vivo. The results of this study highlight the potential of this novel contrast agent for mapping tumor pH when combined with control IBCAs using RpH-MRI.

The accumulation of excess acids in the extracellular space can promote aggressive and treatment-resistant tumor phenotypes [23, 24]. Therefore, a method for assessing the extracellular pH of a tumor would be beneficial for selecting the appropriate therapies. Various techniques have been proposed for measuring the pH in vivo, including magnetic resonance imaging (MRI) [25], magnetic resonance spectroscopy (MRS) [26], and positron emission tomography (PET) [27]. MRI has significant potential for mapping tissue pH in combination with its ability to provide detailed anatomical images and unlimited tissue penetration depth, making it an ideal tool for a variety of applications [28, 29].

The Fe-en-tCDTA iron complex demonstrated notable pH-dependent T1 relaxivity and contrast effects under acidic conditions, a characteristic feature of many tumors, which decreased to approximately one-third at pH 7.4. This property sets Fe-en-tCDTA apart from its counterparts, Fe-DTPA and Fe-tCDTA, which demonstrated consistent T1 relaxivity in the pH range of 5.5–7.4. Fe-tCDTA has a single coordination site for water molecules that can exchange with bulk water, resulting in an efficient inner-sphere relaxation. In contrast, Fe-DTPA lacks inner-sphere water relaxation, which reduces its contrast effect for MRI applications. However, the inner-sphere relaxation of Fe-en-tCDTA is pH dependent. At neutral to high pH values, the terminal amino group is deprotonated and coordinates with the central iron, thereby preventing water coordination. As the pH decreases, the terminal amine group becomes protonated, preventing its coordination with the central iron and allowing water coordination, and therefore, efficient inner-sphere relaxation.

Fe-en-tCDTA was stable and biocompatible both in vitro and in vivo. The physical and chemical stability of Fe-en-tCDTA was confirmed over extended periods with no significant precipitation or degradation, ensuring its practical utility. The biocompatibility of Fe-en-tCDTA was verified through a MTT assay in vitro, which demonstrated minimal cytotoxicity in BRL-3A and 4T1 cell lines at concentrations of up to 20 mM and 24 h incubation time. This plasma concentration corresponds to an application dose of approximately 1 mmol/kg [19]. Moreover, in vivo, the peak concentration of Fe-en-tCDTA was reached after only a few minutes (approximately 3 min). Thereafter, the contrast agents were rapidly eliminated from the blood. The rapid clearance and renal excretion enhance its clinical appeal without causing significant short-term toxicity and reducing the risk of long-term retention and associated toxicity.

Our RpH-MRI approach is feasible and offers important improvements over previous methods [6, 7]. In previous studies, pH mapping in vivo was performed by a similar method using GBCA, GdDOTA-4AmP [8]. The relatively long blood circulation time of this chelate necessitates an extended interval between the injection of the pH sensor and control probe. To compensate for the relatively slow kinetics, the signal baseline after the second injection must be corrected for the first injection by modeling the contrast contribution of the remaining circulating control agent. To improve the tissue biodistribution and kinetic properties of the agent, Paranawithana et al. [12] reduced the overall charge of the modified complexes. However, the pH dependency of r1 of the modified complex (GdDOTA-1AmP) was significantly diminished at magnetic fields typically employed for small-animal imaging (7 T and 9.4 T). In our study, Fe-en-tCDTA was neutral at pH 7.4, eliminating the need for a counterion, which reduced its osmolality. Additionally, comparison of r1 in different solutions and pH conditions revealed that Fe-en-tCDTA had no significant interaction with serum proteins. Thus, Fe-en-tCDTA had smoother tissue biodistribution. Compared to GdDOTA-4AmP, the blood half-lives of Fe-en-tCDTA, Fe-DTPA, and Fe-tCDTA were shorter, allowing for shorter application intervals of 30 min or 1 h without the need for baseline correction modeling. As indicated previously [30, 31], systemic blood pressure can decrease during prolonged anesthesia. However, Fe-en-tCDTA was quickly washed out from the blood, muscle, and liver even when administered as a second injection (after at least 30 min of anesthesia). Only the kidneys had higher T1 signals after DCE-MRI; however, after 24 h, their T1 maps returned to pre-injection levels.

The pH maps generated using the RpH-MRI technique on a pixel-by-pixel basis in the current study demonstrated the heterogeneous nature of tumor pH, with core regions exhibiting a more acidic pH than the periphery. This provides valuable insights into tumor physiology, which is consistent with existing literature [32–35]. Furthermore, the core areas exhibited reduced chelate concentrations, which is likely attributable to inadequate perfusion. Consequently, RpH-MRI can simultaneously provide information regarding both tissue pH distribution and perfusion.

Our study has several limitations. The RpH-MRI approach assumes that both contrast agents (responsive and control) have identical pharmacokinetics. Although the pharmacokinetic profiles of our iron complexes demonstrated similar biodistribution and rapid wash-in and wash-out kinetics for control agents (Fe-DTPA, Fe-tCDTA) and pH sensor agents (Fe-en-tCDTA), they cannot be generalized. Further research could extend the application of RpH-MRI with Fe-en-tCDTA to additional tumor types or other pathological tissues, and explore the potential of pH mapping for monitoring therapeutic interventions. Furthermore, the presented RpH-MRI requires that the tissue does not move during the measurement, which was achieved by tape fixation of the tumors. However, motion-tracking methods can overcome this limitation. Because the TMI was always below 7 min, the imaging time could be significantly reduced. Moreover, we did not use direct ex vivo measurements to test the extracellular pH. Such measurements are difficult and error-prone owing to tissue damage and other disturbances of the in vivo situation. Nevertheless, the calculated pH ranges of the tumors in the rim and core regions were consistent with those reported in the literature [32–35], which provides indirect support for our results.

## Conclusion

This study demonstrated the potential of RpH-MRI with Fe-en-tCDTA for in vivo pH mapping on a voxel-by-voxel basis (0.4 mm × 0.4 mm × 0.4 mm) and showed that the method was sensitive enough to detect differences in pH between core and rim of tumors. Thus, RpH-MRI with a combination of pH-sensitive Fe-en-tCDTA and the control probes Fe-DTPA or Fe-tCDTA offers significant potential for molecular imaging of pH in preclinical research and is a promising candidate for clinical translation.

## Supplementary Information

Below is the link to the electronic supplementary material.

Supplementary file1 (PDF 2218 KB)

Supplementary file2 (IJM 3 KB)

Supplementary file3 (IJM 9 KB)
