# Supplementary material for: High-resolution quantitative mapping of extracellular pH by ratiometric MRI with iron chelates in a tumor mouse model
Source: Radiol Med. 2025 May 20;130(8):1231–42. doi: 10.1007/s11547-025-02020-z (PMC12367910; doi:10.1007/s11547-025-02020-z)
Supplement: Supplementary file 1 — Supplementary file1 (PDF 2218 KB) [file 11547_2025_2020_MOESM1_ESM.pdf]

Supplementary information

Chemicals

Unless otherwise noted, all chemicals were purchased from Sigma-Aldrich Chemie GmbH (Taufkirchen, Germany) and were used without further purification. Deionized water was produced using a Milli-Q A10 system (Millipore, Billerica, MA, USA) and used to prepare all solutions and dispersions. trans-1,2- diaminocyclohexane-N,N,N′,N′-tetraacetic acid monoanhydride (tCDTA-MA) was synthesized according to the literature (18). Iron chelates were synthesized and characterized according to the literature (18, 19).

Synthesis of ethylenediamine-trans-CDTA monoamide (en-tCDTA)

tCDTA-MA (2.74 g, 7.9 mmol) was gradually added to ethylenediamine (18.49 mL, 277 mmol) under nitrogen atmosphere at room temperature for 5 h and then stirred overnight. Excess ethylenediamine was distilled using a rotary evaporator in a water bath at a temperature of 90°C under a pressure of 8 mbar. The resulting slightly yellowish crystals were dissolved in methanol, and after one week, the white sediment was separated by centrifugation at 1000 × g for 5 min at room temperature and washed with methanol (5 times in total). Air drying in an oven at 80°C resulted in 1.94 g of a white powder.

Synthesis of the Iron (III) chelate of en-tCDTA (Fe-en-tCDTA)

en-tCDTA (1.94 g, 4.995 mmol) was added to 4.8 mL of a 1.04 M aqueous iron (III) chloride solution and stirred at 90°C for 2 h. After stirring overnight at room temperature, the mixture was centrifuged at 3000 × g for 3 min, and the supernatant was adjusted to a pH of 3.5 with meglumine powder under stirring. After 2 hours the pH of the mixture was adjusted to a pH of 6.8 with meglumine powder. The solution was centrifuged again for 3 min at 3000 × g and subsequently filtered through a 0.2 µm syringe filter and autoclaved at 120°C and 1 bar for 20 min.

Synthesis of Fe-tCDTA

2.77 g (69.3 mmol) of sodium hydroxide was added to a solution of 13.5 mL (13.8 mmol) of iron (III) chloride hexahydrate very slowly to obtain fresh iron hydroxide. After approximately 5 min, the precipitate was resuspended in water and centrifuged (1000 × g for 3 minutes). The resuspension procedure was repeated three times, and the precipitate was resuspended in 25.4 ml water. Subsequently, 5 g (13.7 mmol) tCDTA was added to the dispersion. The dark brown suspension was stirred (350 rpm) and then heated to 95°C. After approximately 30 min, the color had changed to light yellow. The resulting solution was stirred and heated at 95°C for 3 h. Meglumine powder was added to adjust the pH to 3.5. After 2 h, the pH was adjusted with meglumine powder to 6.8. After incubating overnight at room temperature, the mixture was centrifuged for 3 min at 3000 × g at room temperature, and the supernatant was then filtrated with 0.2 um syringe filter.

Synthesis of the Iron (III) chelate of diethylenetriamine-N,N,N‘,N“,N“-pentaacetic acid (Fe-DTPA)

2.57 g (64.2 mmol) of sodium hydroxide was added to a solution of 12.5 mL of iron (III) chloride hexahydrate (12.8 mmol) very slowly. Fresh iron hydroxide was prepared in the same way as described above and finally resuspended in 20.5 ml water. DTPA (5 g (12.7 mmol)) was added to the dispersion. The dark brown suspension was stirred (350 rpm) and then heated to 95°C. After approximately 20 min, the color had changed to light yellow. The resulting solution was stirred and heated at 95°C for 2 h. Meglumine powder was added to adjust the pH to 3.5 and after 2 h to 6.8. After incubating overnight at room temperature, the mixture was centrifuged for 3 min at 3000 × g at room temperature, and the supernatant was then filtrated with 0.2 um syringe filter. A clear reddish-brown solution was obtained and autoclaved at 120°C and 1 bar for 20 min.

All prepared solutions were tested for iron concentration with 6 N HCl and 0.3% H_2O_2 using the BioTek Gen5 software with absorbance measurement at 410 nm and analyzed by reverse-phase high-performance liquid chromatography (HPLC) on a DIONEX UltiMate 3000 system according to the literature (18).

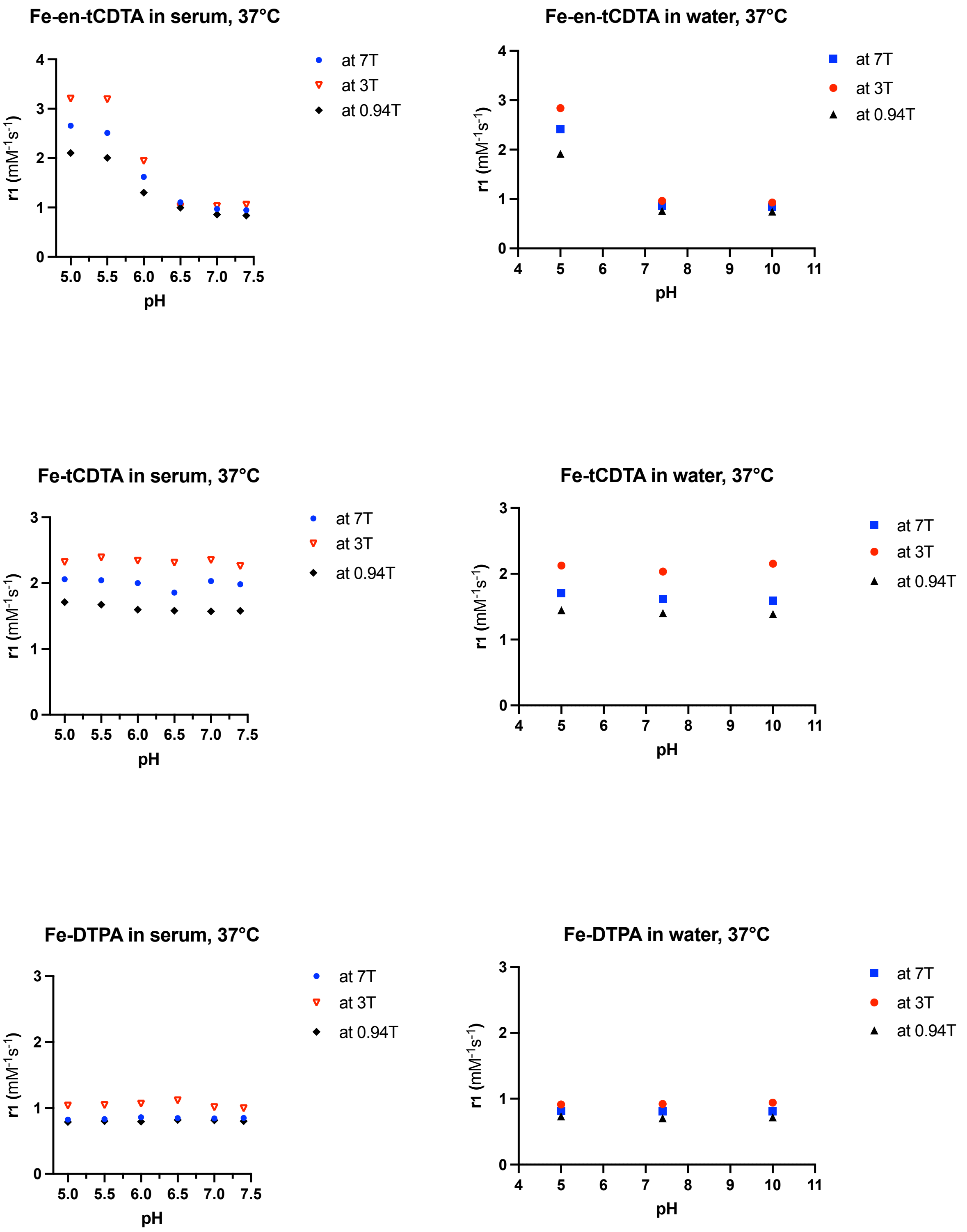

Supp. Figure 1. Effects of field strength on the T1 relaxivity of low-molecular-weight iron compounds.

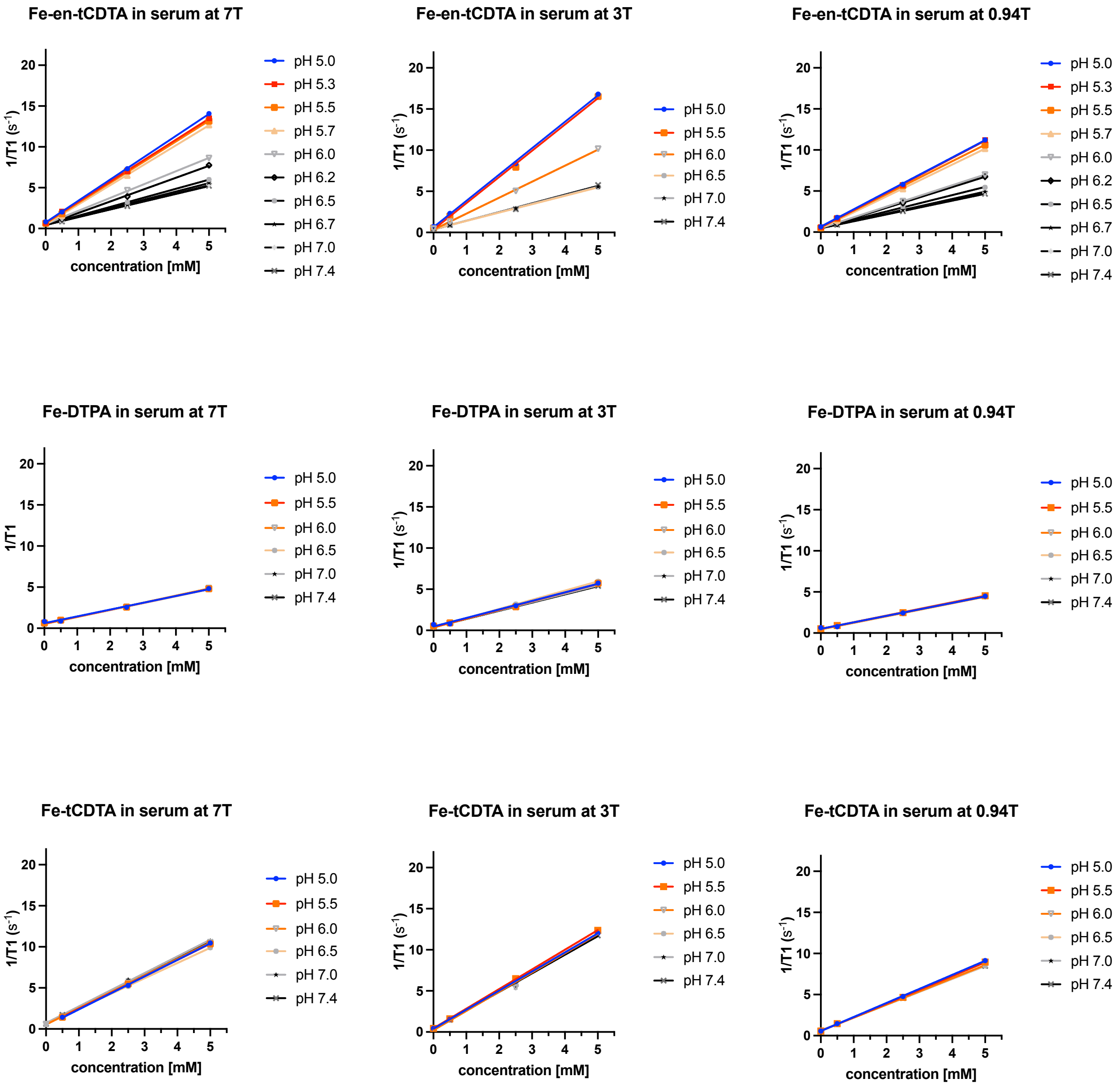

Supp. Figure 2. Relationship between 1/T1 and the concentration of low-molecular-weight iron compounds.

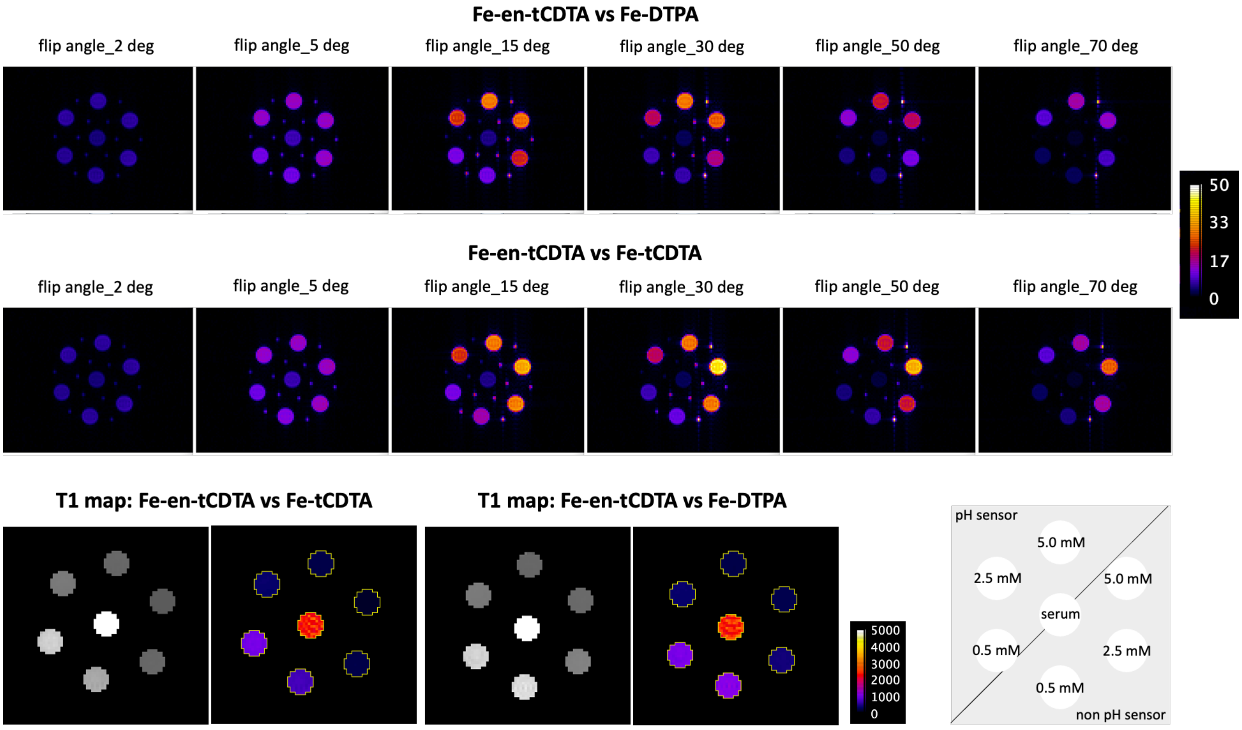

Supp. Figure 3. T1-weighted SPGR images with different flip angles and T1 maps between Fe-en-tCDTA and Fe-DTPA and Fe-en-tCDTA and Fe-tCDTA in vitro, at 7T, pH 7.4, 37°C.

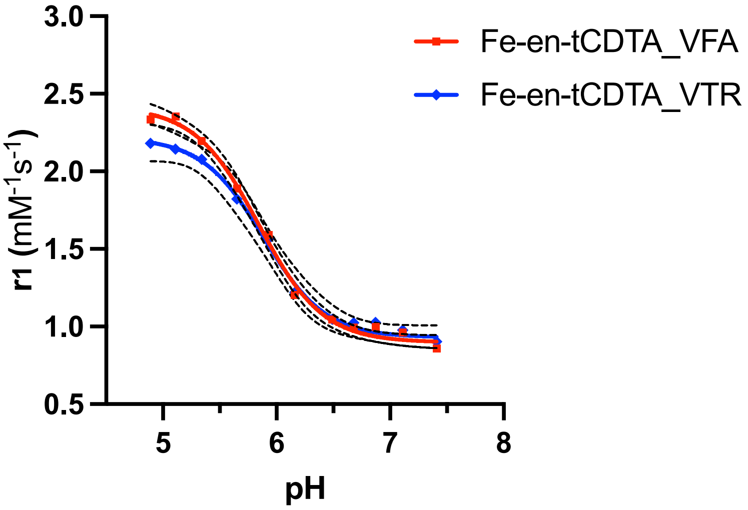

Supp. Figure 4. T1 relaxivity comparison between VFA and VTR pulse sequences in serum at 37°C, 7T. The r1 of Fe-en-tCDTA exhibited more pronounced changes with VFA compared to VTR. VFA=variable flip angle; VTR=variable repetition time.

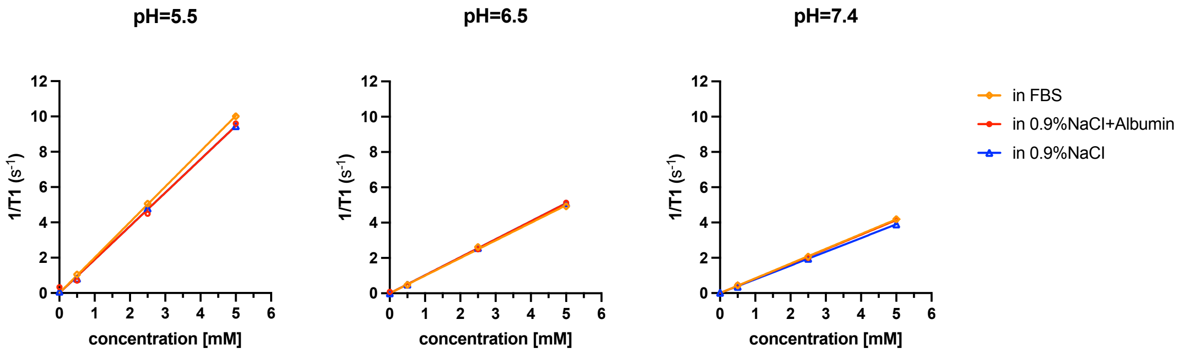

Supp. Figure 5. Comparison of the Fe-en-tCDTA T1 relaxation rates in different solutions at different pH values: albumin concentration: 2.12 g/100mL, same in FBS; FBS: fetal bovine serum; there was no significant difference among different solutions (comparison of all the points (R1 relaxation rates) among different solutions, one-way ANOVA, all p values > 0.99).

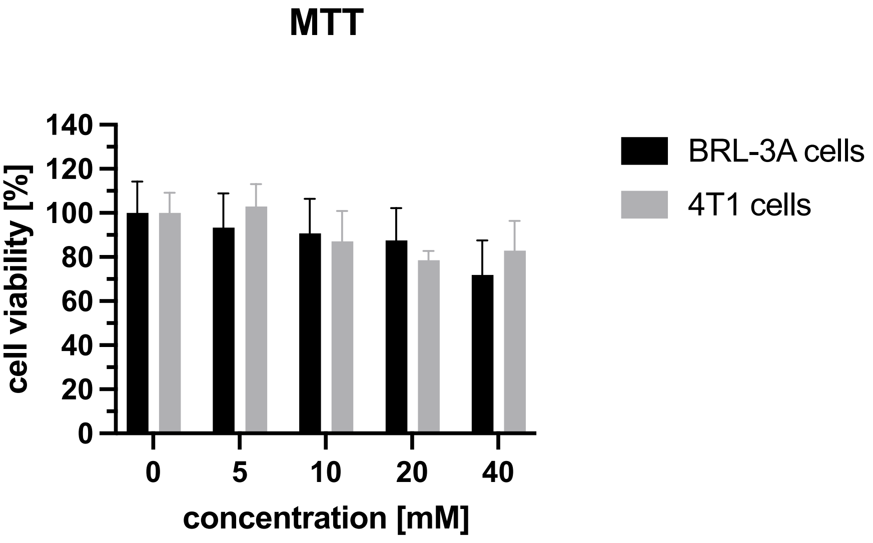

Supp. Figure 6. Relative viability of BRL-3A and 4T1 cells after incubation with Fe-en-tCDTA for 24 h.

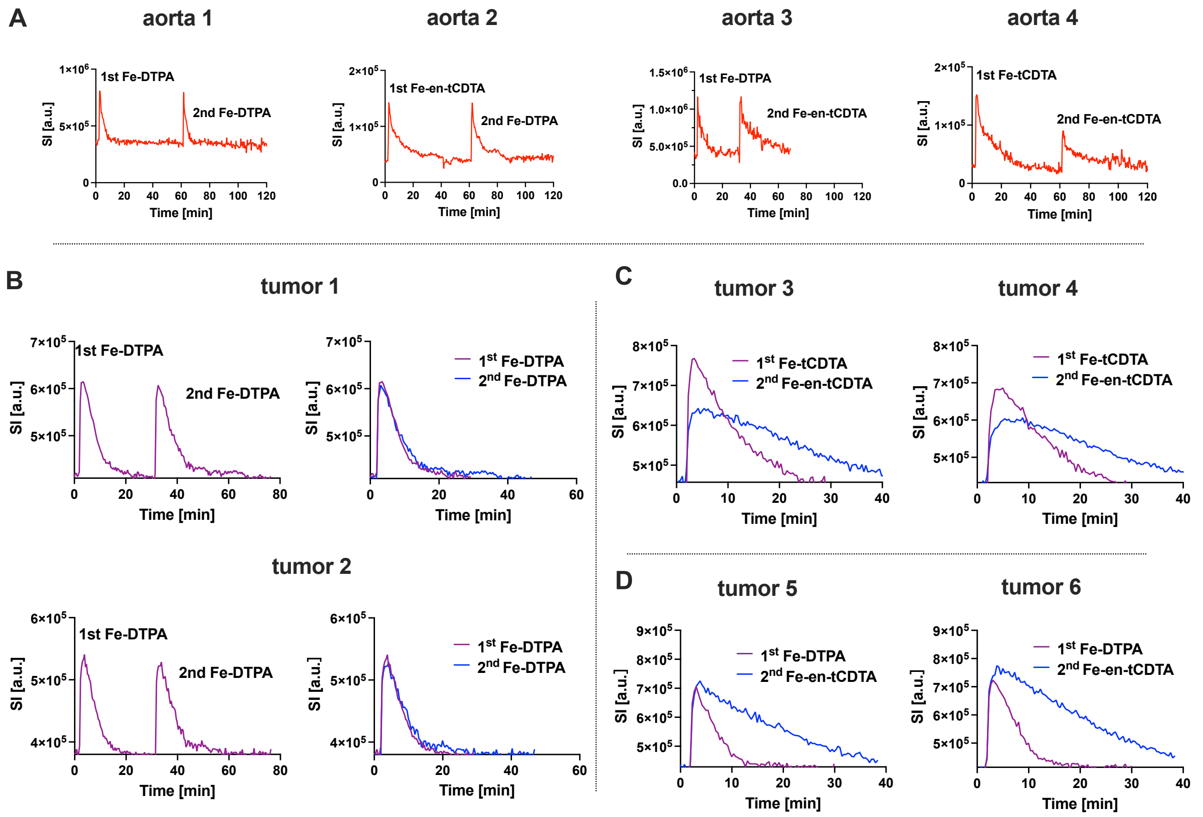

Supp. Figure 7. Preparatory experiments for selection of suitable intervals for RpH-MRI. (**A**) DCE-MRI signal intensity curves of sequentially administered iron-based contrast agents (IBCAs) in the aortas of four different mice, each administered with different combinations. The injections were performed with intervals of 30 min or 60 min. (**B**) DCE curves of sequential injection of two times Fe-DTPA with similar shapes and peaks. (**C**) DCE curves of Fe-tCDTA and the pH-sensor in tumors. (**D**) DCE curves of sequential injection of Fe-DTPA and pH-sensor in tumors. The initial injection doses for these experiments were 0.5 mmol/kg.

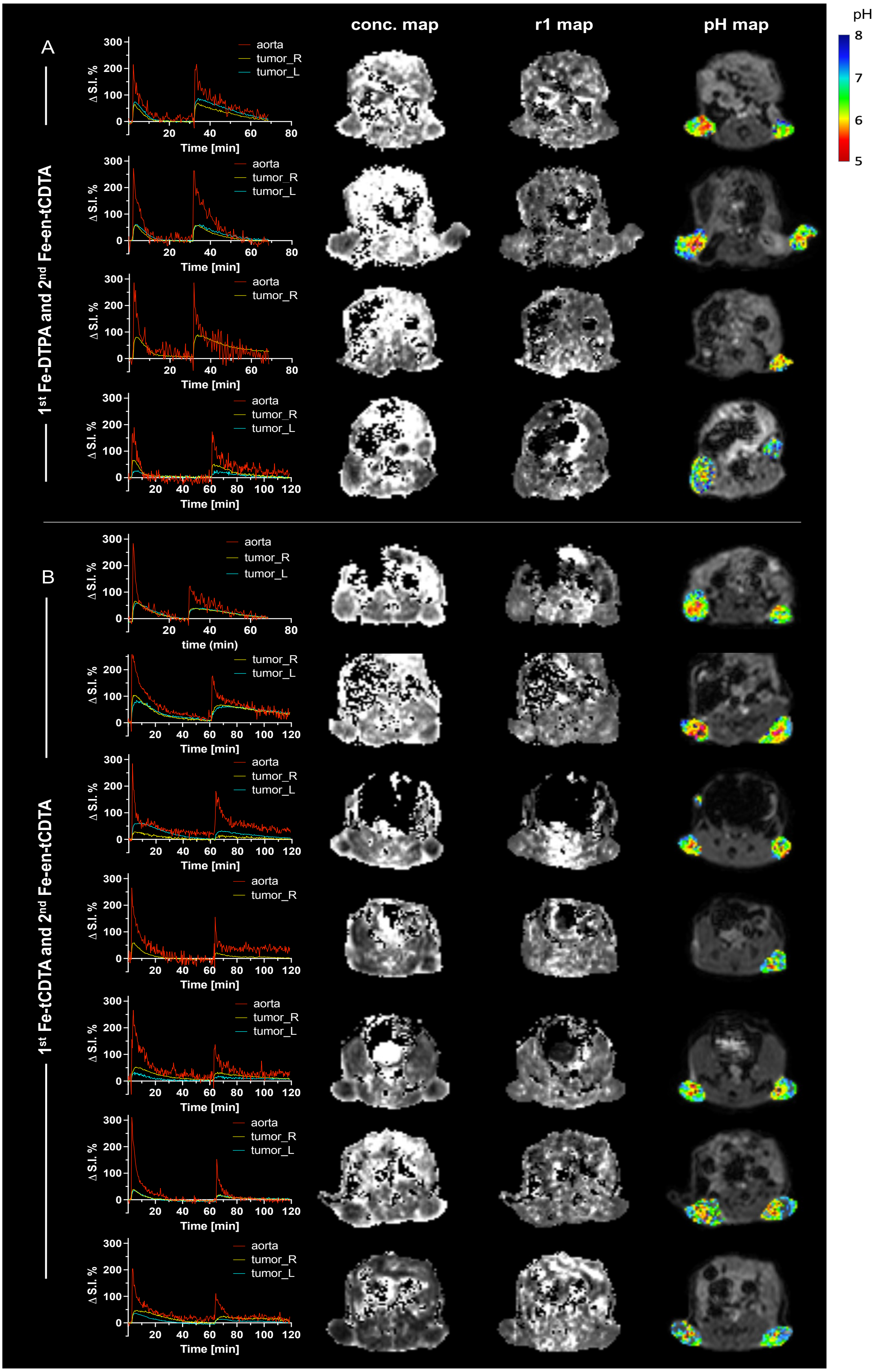

Supp. Figure 8. DCE curves and in vivo mapping. (**A**) Sequential injection of 1st Fe-DTPA and 2nd Fe-en-tCDTA with dose 0.5 mmol/kg. (**B**) Sequential injection 1st Fe-tCDTA and 2nd Fe-en-tCDTA at a dose 0.25 mmol/kg. Twenty tumors from 11 mice were included in the analysis. conc. map: Concentration map.
